# Effect of double adhesive layer application on micro-tensile dentin bond strength of a universal adhesive

**DOI:** 10.3389/fdmed.2024.1484498

**Published:** 2024-10-11

**Authors:** Afnan O. Al-Zain, Asrar Albuqayli, Abdulaziz Albogami, Abdulghani Alkudsi, Meshari Alwabiri, Abdullah T. Koshak, Hattan Alsefri, Eliseu A. Munchow

**Affiliations:** ^1^ Restorative Dentistry Department, Faculty of Dentistry, King Abdulaziz University, Jeddah, Saudi Arabia; ^2^ Advanced Technology Dental Research Laboratory, King Abdulaziz University, Jeddah, Saudi Arabia; ^3^ Ibn Sina School University, Jeddah, Saudi Arabia; ^4^ Faculty of Dentistry, King Abdulaziz University, Jeddah, Saudi Arabia; ^5^ Department of Conservative Dentistry, School of Dentistry, Federal University of Rio Grande do Sul, Porto Alegre-RS, Brazil

**Keywords:** double bonding layer, double bond curing, micro-tensile strength, light curing, universal adhesive, bonding, adhesive, dentin bond strength

## Abstract

**Introduction:**

Achieving optimal dentin bond strength is crucial for the long-term success of adhesive restorations. This study aims to evaluate the impact of double adhesive layer application, with and without light curing between applications, on the micro-tensile dentin bond strength (µ-TBS) of a universal adhesive, in comparison to the conventional single-layer application.

**Methods:**

Intact human molars were divided into three groups (*n* = 15) according to the adhesive application technique using a universal self-etch adhesive (Tetric N-Bond Universal, Ivoclar) as follows: (1) according to the manufacturer's instructions, (2) double-layer application without light curing between layers, and (3) double-layer application with light curing between layers. Samples were immediately tested for µ-TBS, with failure types recorded as adhesive, cohesive, or mixed. Representative samples were observed by scanning electron microscopy. Data were analyzed using multiple-way ANOVA (*α* = 0.05).

**Results:**

The double adhesive layer with and without light curing between layers showed similar μ-TBS to that of the control group (*p* > 0.05).

**Discussion:**

From a clinical perspective, these findings suggest that the accurate application of a single layer of a universal adhesive can be as effective as more complex techniques. Additionally, the use of universal bonding agents may have contributed to the outcomes observed in this study. In conclusion, double adhesive layer application and light curing between adhesive layers did not increase the µ-TBS with the universal adhesive agent explored.

## 1 Introduction

The correct application of dental adhesive systems is an essential step for the longevity of light-activated resin-based dental restorations (1, 2). However, despite the availability of excellent bonding agents, the adhesive layer remains the weakest area of a restoration (3). The overall failure rate of resin-based composite restorations ranges from 10.59% to 13.13% (4), possibly due to bond degradation and microleakage, as the adhesive layer can interact with water and salivary enzymes in the oral environment, causing poor marginal sealing, marginal discoloration, recurrent caries, and, ultimately, loss of restoration retention (5). In addition, adequate polymerization is crucial for optimal material properties in dental restorations (6, 7). Insufficient light curing can result in inadequate polymerization, compromising mechanical properties and reducing material durability (6, 7). Therefore, an appropriate light curing technique is essential when curing the adhesive layer (7–9). Typically, the distance between the light curing unit tip and restoration is approximately 6–8 mm from the cavosurface margins to the bottom of the proximal box (10). Ensuring the correct exposure duration, distance, and angle during curing is crucial for even light distribution and complete polymerization (7, 9).

Self-etch adhesive systems partially remove the smear layer using a not-rinsed-off weak acid, resulting in a smear layer partially dissolved and impregnated within the adhesive. Notably, self-etch adhesives may display higher bond strength on dentin than on enamel, thus highlighting a more convenient protocol than the use of etch-and-rinse adhesives, requiring less chair time, lower technique sensitivity, and resulting in less postoperative sensitivity (11, 12). Universal adhesive systems have been developed to simplify the clinical application steps because they can be applied in self-etch or etch-and-rinse modes (13). Selectively etching the enamel margins with phosphoric acid allows an etch-and-rinse mode on the enamel and a self-etch approach on the dentin, and this application mode may be recommended to increase the bond strength (13). Dentin adhesives show favorable immediate bond strength, although long-term dentine-bonded interfaces deteriorate, resulting in limited durability over time (14). Of note, a previous study reported that the double-layer application of single-step self-etch systems improved the initial bond strength and longevity (15).

Research on the application of double adhesive layers to dentin has yielded conflicting results. Several studies found that double application significantly improved micro-tensile bond strength (µ-TBS) on wet dentin (16) and increased microshear bond strength (17). Double application also enhances resin tag formation and limits voids within the adhesive layer (16). However, some studies reported no significant effect of double application on bond strength (18, 19). Other studies have reported that universal adhesives generally perform better with double or triple applications (5, 20). The double-layer technique may lead to over-etched dentin substrates, contributing to hybrid layer formation with unprotected collagen fibrils (21), possibly resulting in bond strength deterioration over time due to enzymatic degradation (22). Consequently, the double-layer application technique can enhance the bonding characteristics of universal adhesive systems (23, 24).

Studies have explored the impact of applying a double adhesive layer on bond strength, both with and without artificial aging, yielding mixed results. Some research has shown improvements in bonding strength, while others have reported no significant changes in bond strength between single and double adhesive layers (16–18, 25, 26). One study demonstrated that applying a double adhesive layer, with light curing after the second layer, significantly increased bond strength (17). Conversely, another study found no significant difference when light curing was applied to the second adhesive layer (18). One of the protocols investigated was the application of different universal and self-etch adhesives, either in a single layer or two layers, without light curing the first layer (16, 26). In contrast, another study showed that light curing between applications significantly improved bond strength after double-layer application (25). These mixed outcomes indicate that further investigation is needed to explore the impact of light curing each adhesive layer separately—a clinical step that could enhance the quality of polymerization and improve dentin bond strength. This practice has the potential to significantly enhance adhesive performance and the overall success of the restoration, thus making this aspect worth investigating.

This study aims to explore the application of a double adhesive layer with or without light curing each application layer on dentin micro-tensile (µ-TBS) of one universal system compared to a single bond application. The null hypothesis is that no significant difference will exist between the double adhesive layer application with and without light curing each application layer and a single bond application of a universal adhesive on µ-TBS dentin bond strength.

## 2 Materials and methods

### 2.1 Specimen preparation

Ethical approval was obtained from the Research Ethics Committee of King Abdulaziz University Faculty of Dentistry (proposal number: 146-11-19). Intact extracted human molars were collected and stored in formalin (10%) for a minimum of two weeks to ensure disinfection (MMWR 2003) (27). The occlusal surfaces of all teeth were sectioned horizontally using a diamond disc (D-201, Blue Dolphin Products, PTC Company, California, USA) under running water to expose the dentin and create a flat surface. No enamel remained on the surface before the creation of the smear layer. A clinically relevant smear layer was created on the dentin surface using #320-grit SiC paper under running distilled water for 60 s. Teeth were divided randomly into three groups (*n* = 15) according to the adhesive application technique: Group 1 (Gp1), application of the adhesive system according to manufacturer instructions (control); Group 2 (Gp2), double adhesive layer application without light curing between layers; Group 3 (Gp3), double adhesive layer application with light curing between layers.

A universal adhesive (Tetric N-Bond Universal Vivapen, Ivoclar Vivadent, Liechtenstein) was used and applied on the entire cut tooth surface by one investigator using a Vivapen brush. A multiple-emission-peak light-emitting-diode light curing unit (LCU) (Valo Cordless, Ultradent, South Jordan, UT, USA) that has a 10 mm active tip diameter, a power value of 700 mW, irradiance value of 890 mW/cm^2^, and radiant exposure of 8.5 J/cm^2^ was used. For all groups, light curing was performed at a distance of 6 mm between the light curing tip and the adhesive layer to mimic the clinical scenario (28).

**Figure 1:**
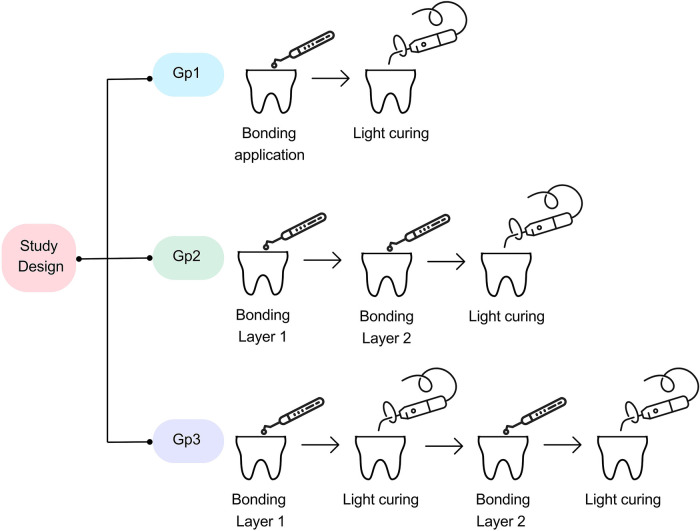
Study design. Gp1: the adhesive layer was applied according to the manufacturer's instructions (control). Gp2: two coats of adhesive were applied according to the manufacturer's instructions without light curing between layers. Gp3: two coats of adhesive were applied according to the manufacturer's instructions and light curing was performed between the adhesive layers.

For Gp1, an adhesive layer was applied according to manufacturer instructions, where the adhesive was applied in a rubbing motion, left for 20 s, followed by gentle air drying, then cured for 10 s. For Gp2, two adhesive coats were applied according to manufacturer instructions without light curing between the layers, where the first adhesive coat was applied according to the manufacturer instructions, and then a second layer was applied, then light cured for 10 s. For Gp3, two adhesive coats were applied according to manufacturer instructions, with each layer light-cured separately, where the first layer was applied, light-cured for 10 s, the second layer is applied, and then light-cured for 10 s. The study design is illustrated in Figure 1.

The specimens were restored by placing a metal matrix band (Palodent circumferential matrix, Dentsply, Connecticut, USA) and a 4-mm-thick layer of nano-hybrid resin-based composite (Tetric N-Ceram Bulk Fill, Ivoclar Vivadent, Liechtenstein) which was built-up and then light-cured according to the manufacturer instructions. Specimens were immersed in distilled water and stored in an incubator (37°C) for 24 h. Table 1 lists the compositions of the adhesive- and resin-based composite used in this study.

**Table 1:** Composition of the adhesive and resin-based composite used in the study.

Material	Component	Percentage (%)
Tetric N-bond universal vivapen	Bis-GMA	25%–50%
	Ethanol	10%–25%
	HEMA	10%–25%
	Phosphonic acid acrylate	10%–25%
	UDMA	≥2.5% ≤10%
	TPO	<2.5%
Tetric N-ceram bulk fill	Bis-GMA	3% ≤ 10%
	UDMA	3% ≤ 10%
	Ytterbium trifluoride	3% ≤ 10%
	Bis-EMA	3% ≤ 10%

Bis-GMA, bisphenol A glycidyl methacrylate; HEMA, 2-hydroxyethyl methacrylate; UDMA, urethane dimethacrylate; TPO, diphenyl (2, 4, 6-trimethylbenzoyl) phosphine oxide; Bis-EMA, ethoxylated bisphenol A glycidyl methacrylate.

### 2.2 Micro-tensile bond strength testing

After 24 h, specimens were affixed to an acrylic block using a cyanoacrylate adhesive (Gorilla Glue Company, Cincinnati, OH, USA) and a heptane-based accelerator (Zapit Accelerator, Suite C, Corona, CA, USA). The specimens were sectioned occluso-gingivally perpendicular to the bonding interface using a precision sectioning machine equipped with a 0.5 mm sectioning disc (TechCut 4 Precision Low-Speed Saw, Allied, East Pacifica Place Rancho Dominguez, CA, USA) under running water. Nine beams of approximately 0.8 × 0.8 mm were harvested from the center of each specimen, with their µ-TBS evaluated using a universal testing machine (EZ Test Universal Tensile Tester EZ-SX; Short Model, Shimadzu Corporation, Kyoto Prefecture, Kyoto, Japan). The crosshead speed was set at 1 mm/min using the following equation:

μ-TBS=F/A

Where: F = force applied at failure (Newtons), A = bonded surface area (mm²).

The result is expressed in megapascals (MPa) (29, 30).

### 2.3 Failure mode

The failure modes at the fracture interfaces were observed under a light microscope and classified as adhesive, cohesive, or mixed. Adhesive failure occurs completely at the adhesive interface with no resin-based composite remnants. Cohesive failure occurs completely within the resin-based composite or the dentin substrate. Mixed failure occurs when the failure happens partially in both the restorative material and the dentin surface, with any proportion of resin-based composite or tooth structure at the interface (16, 31).

Three representative specimens from each group were observed by scanning electron microscopy (SEM) (ZEISS EVO, Carl Zeiss Microscopy, White Plains, NY, USA). The fractured specimens were desiccated for 48 h and mounted onto labeled stubs. The specimens were then sputter-coated with gold for 75 s (Quorum, Quantum Design AG Company, Switzerland) and analyzed using an SEM at 150×–550× magnification.

### 2.4 Statistical analysis

The sample size for this study was determined based on an *a priori* power analysis using G*Power software (version 3.1). A one-way ANOVA was planned to detect a mean effect size (f = 0.25) with an alpha level of 0.05 and a power of 0.80. Based on these parameters, the required sample size was calculated to be 15 specimens per group. This sample size was deemed sufficient to detect statistically significant differences in micro-tensile bond strength between the different adhesive application techniques, assuming a mean effect size.

The µ-TBS was calculated and expressed in megapascal (MPa). Specimens that failed entirely in terms of the cohesiveness of dentin and restoration were not statistically analyzed in this study because they did not represent the exact bond strength at the adhesive layer interface. Comparisons among the groups were performed, with data statistically analyzed using the Kruskal–Wallis test with a significance level of *p* < 0.05. SigmaPlot version 12.0 was used to analyze the data. The failure mode of the fractured specimens was described using a percentage description, and the frequency of each failure was analyzed using the chi-square test (in SPSS version 22.0) to verify its association with the groups tested in the study. The level of significance was set at *p* < 0.05. The reliability and probability of failure of the resin-dentin bonds were analyzed by Weibull analysis: the Weibull modulus (m) and characteristic strength (*σ*0) were obtained with a 95% confidence interval.

## 3 Results

**Figure 2:**
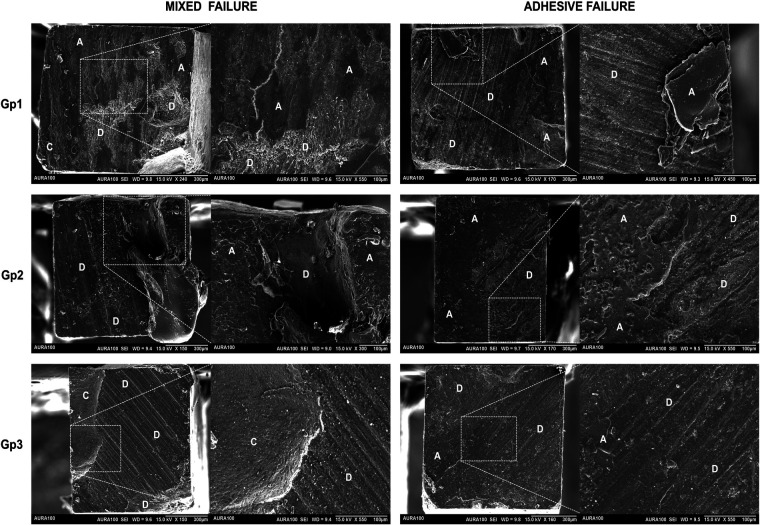
Representative SEM images of the adhesive and mixed failure for the test groups. Gp1: the adhesive layer was applied according to the manufacturer's instructions (control). Gp2: two coats of adhesive were applied according to the manufacturer's instructions without light curing between layers. Gp3: two coats of adhesive were applied according to the manufacturer's instructions and light curing was performed between the adhesive layers. Magnification was 150×, 300×, 450×, and 550×. Differences in magnification were employed to ensure the sample was clear. D, dentin; A, adhesive; C, resin-based composite.

Table 2 lists the results for the bond strength of the tested groups, respective modes of failure of the restored specimens, and Weibull results. The tested groups presented similar median bond strength values with no significant differences among the groups (*p* = 0.235). Concerning the adhesive and mixed modes of failure-only results, the groups presented similar frequencies of adhesive and mixed failures (*P* = 1.000). Weibull analysis demonstrated a similar probability of failure for the three tested groups, which exhibited similar moduli and characteristic strengths (*p* > 0.05). The SEM images show the representative adhesive and mixed failures for the test groups that failed at the interface (Figure 2).

**Table 2:** µ-TBS bond strength results (MPa) for the groups tested in the study.

Parameters	Gp1 (control)	Gp2	Gp3
Mean ± SD	19.8 ± 9.0	23.1 ± 9.9	21.8 ± 10.1
Median (min.—max.)	18.2 (9.4–46.4)	21.4 (10.8–50.1)	20.2 (6.2–51.2)
Mode of failure (%)	A: 55.6/M: 44.4	A: 55.6/M: 44.4	A: 55.6/M: 44.4
*m* (95% CI)	2.7 (2.1–3.4)	2.9 (2.2–3.6)	2.5 (2.0–3.2)
*σ*_0_ (95% CI)	22.2 (19.6–25.1)	23.8 (21.1–26.7)	25.2 (22.0–28.7)
*r* ^2^	0.8589	0.8961	0.9396

No statistically significant differences were found using Kruskal-Wallis test (*p* = 0.235).

Gp1: the adhesive layer was applied according to the manufacturer's instructions (control). Gp2: two coats of adhesive coats were applied according to the manufacturer's instructions without light curing between the layers. Gp3: two coats of adhesive coats were applied according to the manufacturer's instructions, and light curing was performed between the adhesive layers. SD, standard deviation; min., minimum value; max., maximum value; *m*, Weibull modulus; 95% CI: 95% confidence interval; σ_0_, characteristic strength; *r*^2^, correlation index; A, adhesive failure; M, mixed failure. Cohesive failure was excluded because it did not represent an adhesive interface failure.

## 4 Discussion

One of the primary challenges in restorative dentistry is the prevention of bond degradation and microleakage—common issues when adhesive systems interact with salivary enzymes, with the integrity of the adhesive layer being crucial for the longevity of restorations. While the double application of adhesive layers could theoretically provide additional protection by acting as a stress-absorbing layer (19), increasing the thickness of the adhesive layer by applying multiple layers can lead to uneven polymerization and potential solvent entrapment, adversely affecting bond strength, consistent with a previous study that reported that thicker adhesive layers do not necessarily result in stronger bonds (19). Indeed, the potential for occurring solvent entrapment is higher in thicker layers. In our study, although the teeth varied in size, specimens were consistently obtained from the center of each tooth to standardize the location for beam harvesting. A bulk-fill resin-based composite was used, with a height of 4-mm to replicate the typical restoration thickness and ensure sufficient beam height. Prior to harvesting, the specimens were immersed to simulate clinical conditions.

Our study did not show a significant improvement in the bond strength with double adhesive layer application or with light curing of each adhesive layer, indicating that the additional polymerization of each layer did not affect the bond strength or overall quality of the adhesive interface. Our results disagree with those of studies that showed that the multilayer application of universal adhesives improved dentin bond strength (5, 26). The composition of the universal adhesive may have contributed to the findings. The adhesive used in this study contained bisphenol A glycidyl methacrylate (Bis-GMA), urethane dimethacrylate (UDMA), and 2-hydroxyethyl methacrylate (HEMA) monomers, well-known monomers with a good degree of conversion upon light curing. In addition, the adhesive contains diphenyl (2, 4, 6-trimethylbenzoyl) phosphine oxide (TPO) photoinitiator, which is very sensitive to the short wavelength violet light region (7, 9), thus resulting in high microhardness, cross-link density, degree of conversion, and micro-flexural strength for up to 1-mm depth when photocured with a multiple-emission-peak LED unit (32–34). Worth mentioning, since the adhesive layer is typically less than 1-mm thick, it is expected that sufficient polymerization was achieved.

Regarding the mode of failure, the predominance of adhesive failures followed by mixed failures in this study can be explained by the inherent weaknesses at the adhesive interface. Adhesive failure typically occurs when the bond between the adhesive and the tooth structure is lower than the dentin's or resin-based composite restoration's cohesive strength, becoming the weakest link during stress loading (30). This can occur due to incomplete infiltration of the adhesive into the demineralized dentin, as demonstrated in Figure 2, where a scratchy surface is easy to observe, indicating that the failure occurred at the exact interface between the superficial dentin and the applied adhesive. Some reasons that explain this incomplete infiltration are the suboptimal polymerization of the adhesive layer, decreasing the bonding effectiveness of the material to the substrate, or due to the presence of contaminants like moisture or saliva during bonding, although any contamination was carefully minimized by following a standardized protocol of application of adhesive. In turn, mixed failures involve a combination of adhesive and cohesive failures, indicating that while the adhesive bond may have been reasonably strong to withstand mechanical loading, thus causing the failure within the cohesiveness of dentin or within the bulk of the resin-based composite restoration, there were also areas where the bond between the adhesive and the substrate did not resist mechanical stress (35). The occurrence of mixed failures was similar among the tested groups (Table 2), and the SEM micrographs shown in Figure 2 show the clear presence of areas consisting of the resin-based composite restoration, the dentin substrate as an irregular area with deep removal of hydroxyapatite tissue, and the scratchy surface suggestive of adhesive failure.

The occurrence of adhesive failures was higher in the present study and regardless of the number of adhesive layers. One can suggest that the adhesive system used did not create an even bond across all tested specimens, with the weakest link being the bond at the adhesive interface. Notably, the bonding material tested in the present study does not contain the functional monomer 10-methacryloyloxydecyl dihydrogen phosphate (10-MDP), which has been recognized as an important ingredient for dentin bonding (36, 37), probably due to its ability to chemically bind to the hydroxyapatite found in dentin. Other studies have also shown the improved bonding potential of 10-MDP-based adhesives (16, 25). Thus, one should consider that the effects of additional layers of adhesive could be different when using adhesive systems based on 10-MDP, perhaps differing from the present findings. Nevertheless, and despite the composition of the adhesive, it seems critical to ensure optimal adhesive application techniques and ideal polymerization to enhance the integrity of the adhesive bond and reduce the occurrence of adhesive failures. While cohesive failures provide valuable information about bond strength, they were excluded in this study to focus on the bond strength at the adhesive interface rather than the strength of the restorative material itself. However, we investigated with and without cohesive failure, and the results were similar.

The performance of adhesive systems is influenced by several factors, including the chemical composition of the adhesive, the presence of solvents, and the application technique (12). The universal adhesive used in this study, Tetric N-Bond Universal, is a self-etching adhesive containing monomers that can form strong chemical bonds with dentin. The findings of this study indicated that single-layer applications, when performed correctly, were sufficient to achieve optimal bonding. Therefore, the null hypothesis was accepted.

From a clinical perspective, these results reinforce the idea that careful application of a single adhesive layer can be as effective as more complex procedures. Therefore, adhering to the manufacturer's instructions for adhesive applications is generally sufficient to achieve reliable bond strength. Applying a double adhesive layer does not confer additional benefits and may complicate the procedure without improving outcomes, highlighting practical implications for dental practitioners, as it simplifies the restorative process and reduces technique sensitivity. Furthermore, this study emphasized the importance of proper adhesive application techniques. Notably, ensuring adequate air-drying and light curing can prevent solvent entrapment and incomplete polymerization issues—critical for maintaining bond strength and restoration longevity.

Study limitations include tooth variability. Although efforts were made to standardize the location of beam harvesting, natural variations in tooth structure, such as dentin density or mineralization, may have influenced the results. Using teeth with varying sizes may introduce an inconsistency in the bonding surface and may have contributed to the results. Also, the study was a short-term study. The factor of aging long-term storage could provide more insight into the durability of the adhesive bond over time, especially when subjected to a dynamic oral environment, such as temperature changes, mechanical stresses, and enzymatic degradation. In addition, the study only evaluated one type of universal adhesive. The results may differ using other adhesive systems with different chemical compositions, limiting the generalizability of the findings.

Future research should explore the long-term effects of double adhesive layers under varying oral conditions, such as pH fluctuations and mechanical stresses. Additionally, investigating the roles of different adhesive chemistries and their interactions with dentin could provide further insights into optimizing adhesive performance.

## 5 Conclusion

This study concludes that applying a double adhesive layer, with or without light curing between the layers, does not enhance the micro-tensile bond strength when using the tested universal adhesive. These findings suggest that, while double adhesive layers do not enhance bond strength, adhering to the manufacturer's instructions for single-layer applications is effective. This approach simplifies clinical procedures and emphasizes the importance of precise application techniques for achieving adequate dental restorations. Further research could build on these findings to explore new adhesive formulations and application methods to enhance restoration longevity.

## Data Availability

The original contributions presented in the study are included in the article/Supplementary Material, further inquiries can be directed to the corresponding author.
